# Medication management during risk of dehydration: A qualitative study among elderly patients with impaired renal function and informal caregivers

**DOI:** 10.1080/13814788.2024.2413097

**Published:** 2024-10-11

**Authors:** Tristan Coppes, Daphne Philbert, Mijke Van Riet, Teun van Gelder, Marcel Bouvy, Ellen Koster

**Affiliations:** a Department of Pharmacoepidemiology and Clinical Pharmacology, Utrecht Institute for Pharmaceutical Sciences (UIPS), Faculty of Science, Utrecht University, Utrecht, The Netherlands; b Department of Clinical Pharmacy & Toxicology, Leiden University Medical Centre, Leiden, The Netherlands

**Keywords:** Impaired renal function, informal caregivers, dehydration, elderly, sick day guidance

## Abstract

**Background:**

Patients with impaired renal function are at an increased risk of dehydration due to vomiting, diarrhoea or fever (so-called sick days). Temporary medication adjustment during sick days is necessary and current initiatives and information materials for patients are available. However, the knowledge, experiences and information needs of patients and informal caregivers about sick day guidance are unknown.

**Aim:**

To gain insight into the understanding of safe medication use during periods of dehydration risk in elderly patients with impaired renal function and their informal caregivers.

**Design and setting:**

Qualitative interview study with patients with impaired renal function and unrelated informal caregivers from three community pharmacies in the Netherlands.

**Method:**

The interviews were conducted by telephone or live by two researchers in November 2020–September 2021 and audiotaped and transcribed verbatim. The coding of transcripts was performed deductively and inductively in Nvivo 12, a thematic analysis was applied.

**Results:**

In total 12 patients and 11 unrelated informal caregivers were included. Three main themes were derived from the interview guide and subthemes emerged from the transcripts. The included patients and informal caregivers had limited knowledge about medication management during sick days. In contrast to patients, informal caregivers seemed interested in a medication management protocol for sick days.

**Conclusion:**

Patients with impaired renal function and informal caregivers have little knowledge about and experience with dehydration and safe use of medication during sick days. General practitioners and pharmacists should involve the care network, including informal caregivers, when implementing sick day guidance.

## KEY MESSAGES

Elderly patients and informal caregivers seem to have little knowledge about medication adjustments during sick days or heatwaves

Informal caregivers appear to be more interested than patients in information about medication management on sick days

GPs should involve informal caregivers in providing information on medication management during risk of dehydration.

## Introduction

One out of ten avoidable medication-related hospital admissions in the Netherlands results from not adjusting medication in patients with impaired renal function [1]. Elderly patients with impaired renal function are at increased risk of acute kidney injury (AKI) due to hypovolemia, especially during periods with a risk of dehydration [2], such as vomiting, diarrhoea, fever (so-called ‘sick days’) or high environmental temperatures. Many of these, mostly elderly, patients use diuretics and angiotensin-converting-enzyme (ACE) inhibitors [3,4]. Ongoing use of these medications during sick days and heatwaves, combined with a decreased thirst sensation in the elderly, could lead to the development of AKI [5,6] which often results in hospital admission [7].

To prevent adverse events during sick days and heatwaves, patients with impaired renal function and their (informal) caregivers must be aware of the risk of continued medication use during periods with a risk of dehydration. Currently, evidence shows that the knowledge of patients is low and patients are often not informed about sick day management [8]. Therefore, providing patients with information and instructions regarding temporary medication adjustment is necessary. This can for example be done by GPs during prescription or by pharmacies during dispensing of medication. The pharmacists’ role is to promote the safe use of medication, however, they are likely not aware when patients are sick because patients would contact their GP. Therefore, the current role of pharmacists is limited to information provision to patients.

Several guidelines and recommendations have been developed in, for example, the United Kingdom [9,10] and Scotland where they released a ‘Medicines Sick Day Rules Card’ with instructions on when and how to adjust medication [11]. In the Netherlands, the Dutch general practitioner (GP) guidelines on chronic kidney disease suggests GPs to consider temporarily adjusting certain medications during periods with increased risk of dehydration [12]. In addition, the Dutch Kidney Foundation published a brochure with instructions during sick days for patients [13]. However, these initiatives have not been evaluated and their effectiveness has not been studied yet. Thus far, it is unknown whether patients or informal caregivers are receiving any information from their GPs or pharmacists about the risk of dehydration during sick days and the included temporary medication adjustments. Alignment between healthcare professionals about implementing sick day guidance seems to be difficult [14]. Research is needed to study this possible knowledge and information provision gap and to align patient education and counselling materials with the needs and wishes of patients and informal caregivers.

Therefore, this study aimed to gain insight into the current knowledge, information needs and experiences of elderly patients with impaired renal function and their informal caregivers about safe medication use during periods with increased risk of dehydration.

## Methods

### Study design and setting

Semi-structured interviews with patients with impaired renal function and informal caregivers were conducted between October–November 2020 and September 2021. Patients and informal caregivers were recruited from three community pharmacies in two small villages (approximately 10.000 inhabitants) close to two cities in the centre of the Netherlands. The pharmacies were selected based on existing contacts and were located close to the researchers as COVID restrictions were still present and travelling was difficult. This study was conducted in compliance with the requirements of the Institutional Review Board of the division Pharmacoepidemiology & Clinical Pharmacology, Utrecht University and with the help of pharmacists belonging to the Utrecht Pharmacy Practice network for Education and Research (UPPER) [15] (reference number UPF2008).

### Study population

Patients were included based on the criteria as shown in Table 1 [11,13]. Informal caregivers were approached to participate if they were registered as informal caregiver in the pharmacy information system and if the patients they were caring for met the inclusion criteria. Informal caregivers were excluded if the patient whom they were caring for was already participating in this study. The selection criteria were applied to the electronic information system of the pharmacy. Eligible patients and informal caregivers were approached by telephone from their community pharmacy by an employee or a researcher who signed a confidentiality agreement. If they agreed to participate, the research team would send the participant an information leaflet about the study and the interview was planned. Data on response before inclusion was not collected. Before the start of the interview, the participant was asked to give informed consent and permission to audio record the interview.

**Table 1.** Inclusion criteria of patients and informal caregivers.

Inclusion criteria^1^
Age ≥65 years
Chronically using ≥5 medications
Existing impaired renal function < 60 mL/min/1.73m^2^
Using a multidose medication dispensing system
At least one prescription for high-risk medication in the past three months^2^

^1^ The same inclusion criteria were applied to the patients that the informal caregivers were caring for.

^2^ High-risk medication included angiotensin-converting enzyme (ACE) inhibitors, angiotensin receptor blockers (ARBs), diuretics, NSAIDs, sodium-glucose co-transporter-2 (SGLT2) inhibitors and metformin.

### Data collection

The participants could choose whether the interview was conducted by telephone, video-calling or face-to-face in the pharmacy. The interviews were conducted by two researchers individually (MR and TC). The interview guide was developed based on the information and research gaps that were identified by the researchers in literature and experiences from daily practice. The guide consisted of four main topics: patient knowledge of impaired renal function; information received from healthcare professionals; medication use behaviour during risk of dehydration; and informational needs of the patient (Appendix 1). The definition of a ‘sick day’ was explained to the participant beforehand. For the interviews with informal caregivers, the same interview guide was used with minor rephrasing to make sure the questions were fitted to the informal caregiver. The interview guides were evaluated after the first two interviews to improve clarification for the participants. All interviews were audio recorded and field notes were taken during the interview. Data regarding patient characteristics was collected from the pharmacy information system, including gender, age, number of chronic medications in use and most recent available eGFR. Data saturation was reached if no new codes were added for three interviews in a row.

### Data analysis

All audio recordings were transcribed verbatim. The transcriptions were pseudonymised by assigning a study number. Data analysis was a combination of deductive and inductive coding. An initial codebook based on the interview guides was made before coding the first transcripts. During coding new codes were added to the codebook (Appendix 2). The coding of the transcripts was divided between two researchers (MR and TC). With the first 3 interviews, another researcher (EK) was involved and a consensus meeting was held to discuss coding discrepancies between the researchers. Hereafter, TC and MR independently continued coding the remaining transcripts. If a researcher was unsure about a specific coding in the remaining transcripts, both researchers would decide together. Nvivo version 12 software was used during data analysis. After coding, the main themes were derived from the interview guide and subthemes were derived from the data. The themes are described in the result section with illustrative quotes. Reporting of the methods and data is according to the COREQ guidelines [16] (Appendix 3) and standards for reporting qualitative research [17].

## Results

### Participant characteristics

A total of 12 patients and 11 informal caregivers were interviewed. Data saturation was reached because no new codes were added in the last 3 interviews. Most patients (75%) were women, and all informal caregivers were women. The median age of patients was 79 years (Table 2).

**Table 2.** Characteristics of the study population.

	Patients (*n* = 12)	Informal caregivers (*n* = 11)
Female sex, n (%)	9 (75)	11 (100)
Age, median years (IQR)	79 (74–84)	Not collected
Number of chronic medications in use, n (IQR)	9.5 (7–12)	Not applicable
Most recent eGFR, ml/min/1.73m^2^ (IQR)	43 (39–51)	Not applicable

IQR: Inter quartile range, eGFR: estimated glomerular filtration rate.

**Figure 1.**
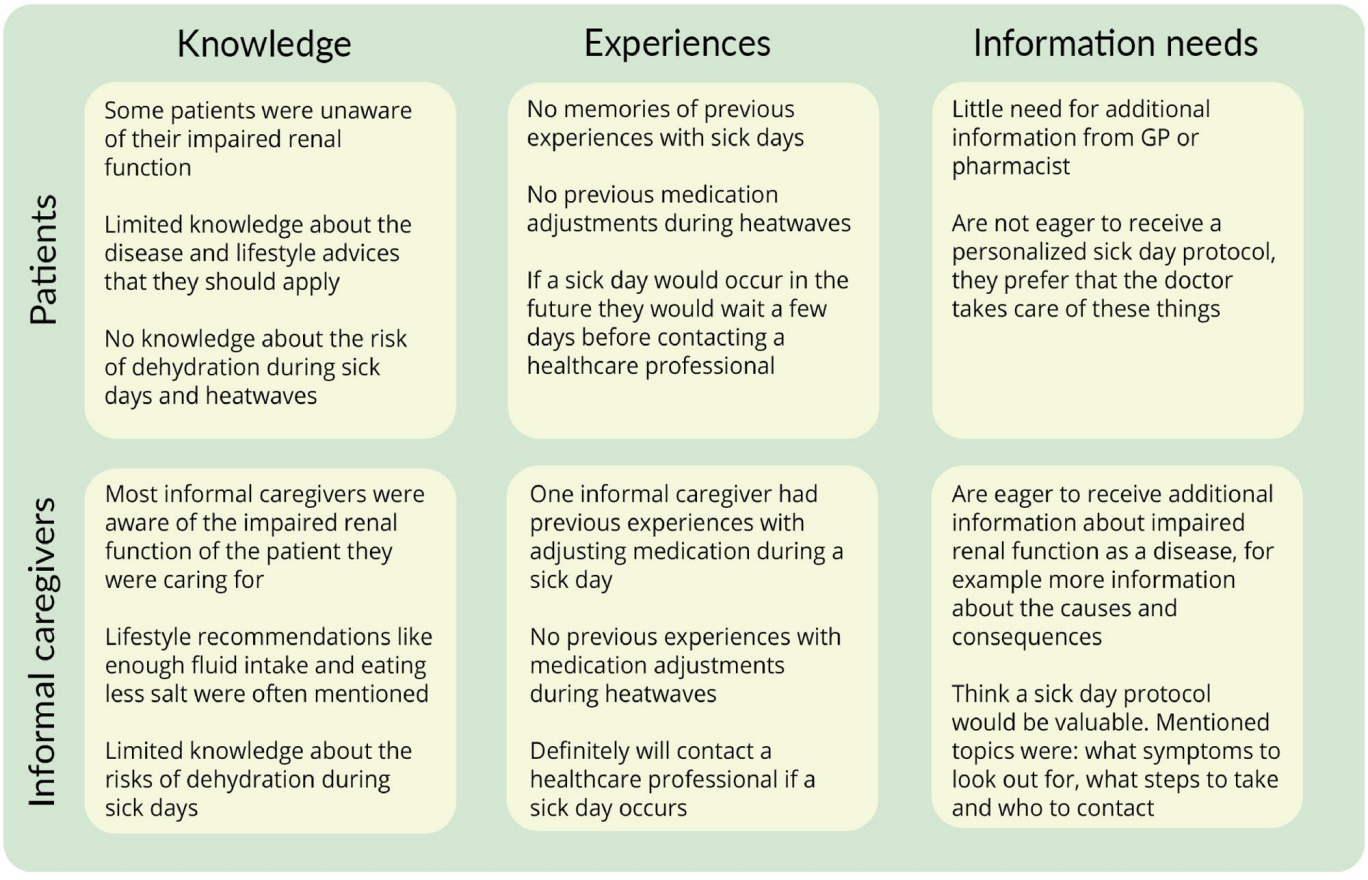
A summary overview of the three main themes and related topics that were mentioned by the patients and informal caregivers. (GP: General practitioner).

Three main topics were derived from the interview guide and used as main themes to structure the results of this study: (1) knowledge of impaired renal function, (2) previous experiences with medication adjustments during dehydration and (3) information needs. All themes will be discussed from the patients’ and informal caregivers’ perspectives (Figure 1).

## Theme 1: Knowledge of impaired renal function

### Patient perspective

The patients who were unaware of their impaired renal function were unsure why they were invited for the interview and also had limited knowledge about what having an impaired renal function entails. The patients aware of their impaired renal function had more knowledge and mentioned general lifestyle recommendations regarding water intake and salt consumption. Some patients mentioned struggling with the application of these recommendations in daily life because they are not thirsty or frequently have to visit the bathroom when their fluid intake increases. In addition, conflicting recommendations existed because patients received instructions to drink more during heatwaves but at the same time had a fluid intake restriction for heart failure.

Yes, that I should drink a lot. I stick to that advice (Female patient (FP) 06 69yr)

I should stick to the rules, I should not drink too much and I should not eat too much salt. (FP01 76yr, also diagnosed with heart failure)

### Informal caregivers perspective

In contrast with the patients, informal caregivers were aware of the impaired renal function of the person they were caring for. Most of the informal caregivers knew about common lifestyle recommendations for patients with impaired renal function. There was, however, little to no knowledge in both patients and informal caregivers about the risk of dehydration during sick days, and of the role the medication played in this regard. A number of informal caregivers brought up that they had received some information years ago but that they don’t remember what it was and that no additional information was recently provided.

No, no additional education or counselling. You know, he has been taking the same medication for years. The prescriptions are just repeated without further notice and if nothing changes in terms of lab results then it simply continues the way it goes. (Informal caregiver [IC] 11)

## Theme 2: Previous experiences with medication adjustments during dehydration

### Patient perspective

Patients had no memory of adjusting medication during a sick day before. Vomiting, diarrhoea or high fever were rarely experienced and patients stated that they would simply take their medication as prescribed during sick days. Some patients mentioned that they would keep an eye on the situation, and would only contact a healthcare professional if the period of sickness continued for more than a few days. A patient mentioned that changing his medication during a sick day would never come to mind.

Honestly, if I would be some kind of sick, I would never think about my medication. And I would never think that I should do something different with my medication (MP08, 84yr)

However, patients were aware of the risk of dehydration during heatwaves. The patients mentioned the advice to drink water during heatwaves regularly but none mentioned adjusting their medication during these periods.

### Informal caregiver perspective

There was one informal caregiver who experienced a sick day period with her mother. During that sick day, the diuretics were temporarily discontinued by the GP. Nevertheless, the patient was eventually admitted to the hospital due to dehydration and delirium. To prevent dehydration in the future, homecare nurses were called in from then on to take care of the patient.

Yes, a year or two ago she [her mother] had horrible diarrhoea. And then they said she was not allowed to take her diuretics anymore because then you are already losing a lot of fluid and that poses high risks for the kidney (IC04)

Almost all informal caregivers stated they would contact a healthcare professional in case of a sick day. An informal caregiver also mentioned that she would call the GP in case of a sick day, but her reasoning was not due to a risk of dehydration. She would be worried that the medication would not work in case of diarrhoea, due to vomiting or rapid GI transit and loss in stool before it had been absorbed from the gut.

Well, if he [husband whom the informal caregiver is caring for] is having diarrhoea for a day or two and is also vomiting then I will definitely call the doctor because I want to know what we are going to do. Because I would be afraid that his medication would no longer work (IC01)

When a healthcare provider needs to be consulted about a sick day, patients and informal caregivers prefer the GP instead of the pharmacist.

Well, I think, the pharmacist is not the one who adjusts the medication, that is the job of the GP. So I do not think it is useful to call the pharmacist (IC10)

## Theme 3: Information needs

### Patient perspective

Patients were asked whether they would like to receive a medication management protocol including additional information on their impaired renal function, the risks of sick days and how to manage medication intake during sick days. The majority of the patients did not need additional information on these topics. This had multiple reasons. Firstly, some patients felt they were too old to learn these kinds of things. Other patients mentioned that they trusted their GP and thought that if the information is necessary for them to know, the GP would have told them already. In addition, they thought that the GP would not prescribe something that could potentially hurt them.

No, I assume, the GP is the expert, he will know what is right for me (FP06 69yr)

Well, no, I will leave that to the GP in all cases (FP13 75yr)

### Informal caregiver perspective

In contrast to patients, informal caregivers were often interested in additional information about, for example, general knowledge about impaired renal function. An informal caregiver mentioned that it would be more valuable to inform her than the patient she is caring for because the patient would not understand the information. The informal caregivers would like to receive a protocol about medication management during sick days; except one caregiver who stated that she did not want to complicate things more and would prefer no extra information. She would just contact the GP if something is wrong. Topics that were mentioned by informal caregivers to be included in such a protocol were how to detect a sick day, what advice should be applied and when to contact a healthcare professional.

Yes, I think that would be wise, then you do not have to sit and wait. Then you have a base to work with and you can decide what to do. [Interviewer: on what topic would like to receive information about?] Information about what symptoms I need to be aware of, what the influence of the medication can be on the symptoms and also when I need to contact a healthcare professional (IC01)

## Discussion

### Main findings

This study suggest that a substantial proportion of the patients with impaired renal function may not be aware of their condition. In addition, many patients and informal caregivers may be unaware of the risk of dehydration during vomiting, diarrhoea, fever or heatwaves and of the safe medication use guidelines that come with these situations. Informal caregivers more frequently had additional information needs compared to patients.

### Strengths and limitations

A strength of this study is that both patients and informal caregivers were interviewed. As the population is ageing and informal caregivers are increasingly involved in the care of patients now and in the future [18,19], involving informal caregivers in the education and counselling process is necessary for implementation of sick day guidance. Another strength of this study was the qualitative design, which allows the researcher to elaborate on certain topics during the interview if necessary.

A limitation of this study was that no agreements were made beforehand about when data saturation was reached. During the interview and coding process, many similarities between participants were observed. Saturation of the data was confirmed because no new codes were added during the last three interviews. Another limitation was the fact that due to the COVID pandemic, most interviews were performed via telephone. Therefore, body language could not be observed and clues might have been missed. In addition to this, a lack of discussion of emotions was also a limitation. Patients did not have much experience with temporary medication adjustments and did not have many information needs, therefore this became difficult to discuss. However we could have focussed more on emotions. Thirdly, the interviews were conducted by two separate researchers which can introduce interviewer bias. Although this was reduced as much as possible by adhering to the semi-structured interview protocol and by reviewing performed interviews. Lastly, limited demographic and contextual information was gathered about the informal caregivers.

### Comparison with existing literature

Our study indicates that the knowledge and awareness about the risk of dehydration in elderly patients with polypharmacy and impaired renal function is low. Similar results were found by Siew et al. [20] about the awareness of the condition and lack of understanding of risk factors in younger patients, median age 54, who were previously hospitalised with moderate to severe AKI.

Although informal caregivers were often familiar with the impaired renal function of the patients they cared for, they had little knowledge of the potential risks during sick days and potential actions during sick days. The informal caregivers were, in contrast to patients, interested in more general information about renal dysfunction and prognosis. This is in line with recent systematic reviews on the information needs of informal caregivers of older adults with chronic health conditions [21,22]

### Implications for research and practice

In this study, the patients showed little interest in a personalised sick day protocol to use at home in case they experience a sick day. The patients mentioned two main reasons for this. Firstly, they do not want to take responsibility themselves, but trust the GP will take care of changes in their medication if necessary. A similar response was also found by Morris et al. [14]. Secondly, their impaired memory and cognitive function made it hard to remember new information, which made them less eager to learn. This can partially be explained by the fact that it is difficult for patients to see the benefit of a sick day protocol because they do not yet understand the importance of medication cessation during sick days. In addition, it was expected that patients with a multidose medication dispensing system would be less willing or capable of taking responsibility for the medication they use [23]. Prior research has shown that these patient characteristics also pose an increased risk of dehydration [24–26]. This underlines the importance of repeatedly providing tailored information to this specific patient group at an early stage of impaired renal function. For example through the same oral and written education during GP visits and pharmacy visits. But even more important is the involvement of informal caregivers and homecare nurses for this group of patients. This is strengthened by the finding of this study that the included informal caregivers were willing to participate more in the care of the patient. They found such tools as a sick day protocol to be a valuable addition to their responsibilities as informal caregivers.

To support the implementation of sick day guidance in practice, healthcare providers should inform all patients about their impaired renal function, as for half of the patients included in this study, this was not known. In addition, sick day guidance information should be tailored to the patient’s situation and should be concise and understandable. A possible solution is to only instruct patients to contact a healthcare professional or informal caregiver in case of vomiting, diarrhoea or high fever. This way, the only action to be taken by the patients is contacting a healthcare provider. This ensures that patients do not receive too much information and that the responsibility for possible medication adjustments lies with the healthcare providers and not with the patient. This could be performed by a GP or practice nurse during prescription but there is also an important role for the pharmacist to repeat the information at dispensing or during medication reviews with high-risk patients. This may be especially important during repeat dispensing as patients often get refill prescriptions without GP visits. Additionally, pharmacists could, with clear agreements with GPs, also provide patients with sick day medication guidance. However, they need to be notified during sick days to be able to provide tailored advice.

For the informal caregivers, more elaborate information can be provided [27]. Besides additional information about impaired kidney function and prognosis, they can, for example, receive a sick day protocol. The protocol should contain information about what symptoms to keep an eye on when to contact a healthcare provider and what general advice to apply as long as the patient is sick. Some informal caregivers could be provided with specific instructions about medication adjustments during sick days, although this should be discussed between the GP and the informal caregiver beforehand.

Another key player in the care network of frail patients that definitely should not be forgotten, is the homecare nurse because they regularly visit the patient at home. Several informal caregivers mentioned their relationship with the homecare nurses of the patients to be very valuable. Homecare nurses should be included in the implementation of sick day guidance. They can fulfil an important role in signalling sick days and often are involved in the medication management of the patient [28,29].

Finally, research should focus on implementation of tailored sick day guidance information in first-line care. The focus should not only be on whether the information is comprehensible and feasible but also on how to reach this specific patient group and their informal caregivers, how to ensure that information is retained and whether sick days are timely being reported to the healthcare professionals.

## Conclusion

In conclusion, elderly patients with impaired renal function and informal caregivers seem to have little experience with adjusting medication during sick days or heatwaves. While patients tend to not express a need for additional information materials, informal caregivers appear to have an interest in additional information. Healthcare professionals, including GPs, pharmacists and practice nurses should address this by offering regular counselling and providing tailored information to both patients and informal caregivers.

## Supplementary Material

Supplemental Material
