# Supplementary material for: Medication management during risk of dehydration: A qualitative study among elderly patients with impaired renal function and informal caregivers
Source: Eur J Gen Pract. 2024 Oct 11;30(1):2413097. doi: 10.1080/13814788.2024.2413097 (PMC11486018; doi:10.1080/13814788.2024.2413097)

# Appendix 1: Interview guide

**[General introduction]**

**[Informed consent]**

**Knowledge of impaired renal function**

1. You are taking a number of different chronic medications which you receive in a multidose dispensing system. Do you get any help with taking your medication?
   - If so, from whom?
   - What is their role?
2. I would like to know more about your impaired renal function. Are you aware of the fact that you have an impaired renal function?
   - How did you find out you had an impaired renal function?
   - What do you notice from your impaired renal function?
   - What do you take into account in your daily life regarding your impaired renal function?

**Information received from healthcare professionals**

1. What information did you receive about impaired renal function when you were diagnosed?

- What did they tell you?
- What do you think about the information they gave you?
- How did you receive this information? (oral, information leaflets?)
- Was the information given sufficient?

If not; why?

- Do you feel you have been sufficiently informed?
- Did you search for any information yourself?

If so: what did you search for? And how?

**Medication use behaviour during risk of dehydration**

1. Patients with impaired renal function must be extra careful with the use of certain medication in some situation. Have you ever been in a situation where you had to be extra careful with your medication?
   - What does this situation look like?

[Explanation from the interviewer about the risk of dehydration, sick days and medication management]

1. Have you ever experienced sick days before?

- What did that situation look like?
- What did you do with your medication?
- Who did you involve in the situation?
- If the patient has not yet experienced this: What would you do if you were in such a situation?
- Have you received information about this topic before?
  - From whom?
  - What did the information include?

1. In case of a sick day, have you ever adjusted your medication?

- Have you ever adjusted your medication due to a heat wave?
- Have you discussed this adjustment with a healthcare provider beforehand?
- Why or why not?

**Informational needs**

1. We want to improve the information that the doctor or pharmacist gives to patients with impaired renal function about the safe use of medication. For example by providing a step by step plan for what to do in case of fever or vomiting. What do you think about such a protocol?

- About what aspects would you like to receive additional information?
- How would you like to receive this information? (Oral, written?)
- From whom would you like to receive this information?
- What other topics would you like to receive information about regarding impaired renal function?

**[Provide short summary, anything to add? End of interview]**

# Appendix 2: Consolidated criteria for reporting qualitative studies (COREQ): 32-item checklist

| No | Item | Guide questions/description | Check? |
| --- | --- | --- | --- |
| **Domain 1: Research team and reflexivity** | | | |
| Personal Characteristics | | | |
| 1. | Interviewer/  facilitator | Which author/s conducted the interviews? | TC and MR |
| 2. | Credentials | What were the researcher's credentials? E.g. PhD, MD | TC is PharmD  MR is BSc |
| 3. | Occupation | What was their occupation at the time of the study? | TC PhD candidate  MR master Pharmacy research student |
| 4. | Gender | Was the researcher male or female? | TC is male MR is female |
| 5. | Experience and training | What experience or training did the researcher have? | TC: training in qualitative interviewing  MR: Unexperienced |
| Relationship with participants | | | |
| 6. | Relationship established | Was a relationship established prior to study commencement? | No |
| 7. | Participant knowledge of the interviewer | What did the participants know about the researcher? e.g. personal goals, reasons for doing the research | Participants were informed about the research by invitation letter including information about the researchers and the research |
| 8. | Interviewer characteristics | What characteristics were reported about the interviewer/facilitator? e.g. Bias, assumptions, reasons and interests in the research topic | The researcher introduced him or herself at the start of the interview. The reasons for, and interests in the research topic were explained to the participants. |
| **Domain 2: study design** | | | |
| Theoretical framework | | | |
| 9. | Methodological orientation and Theory | What methodological orientation was stated to underpin the study? e.g. grounded theory, discourse analysis, ethnography, phenomenology, content analysis | A thematic content analysis was applied. A combination of deductive coding (based on interview content) and inductive coding was used. |
| Participant selection | | | |
| 10. | Sampling | How were participants selected? e.g. purposive, convenience, consecutive, snowball | Participants were selected based on the inclusion criteria applied in the pharmacy's electronic information system. From this list, patients were randomly approached by their pharmacist, a pharmacy employee or researchers based on the preference of the pharmacy |
| 11. | Method of approach | How were participants approached? e.g. face-to-face, telephone, mail, email | Participants were approached in the pharmacy through telephone |
| 12. | Sample size | How many participants were in the study? | 23. Data about response before inclusion was not collected. |
| 13. | Non-participation | How many people refused to participate or dropped out? Reasons? | No participants dropped out after inclusion. |
| Setting | | | |
| 14. | Setting of data collection | Where was the data collected? *e*.g. home, clinic, workplace | The data was collected in the pharmacy or via video. |
| 15. | Presence of non-participants | Was anyone else present besides the participants and researchers? | No |
| 16. | Description of sample | What are the important characteristics of the sample? e.g. demographic data, date | The patients who participated were relatively old and had multiple comorbidities. The informal caregivers were all women. |
| Data collection | | | |
| 17. | Interview guide | Were questions, prompts, guides provided by the authors? Was it pilot tested? | The interview was evaluated after the first two interviews by the research team including a senior researcher. Small adjustments were made before proceeding with the remaining interviews |
| 18. | Repeat interviews | Were repeat interviews carried out? If yes, how many? | No |
| 19. | Audio/visual recording | Did the research use audio or visual recording to collect the data? | Yes, an audio recording was used to collect the data. |
| 20. | Field notes | Were field notes made during and/or after the interview? | Yes, during the interview |
| 21. | Duration | What was the duration of the interviews? | The duration of the interviews was between 20 and 45 minutes. |
| 22. | Data saturation | Was data saturation discussed? | Data saturation was not discussed beforehand. Saturation was looked at in hindsight by evaluating the coding of transcripts. During the last 3 transcripts, no new codes were added. |
| 23. | Transcripts returned | Were transcripts returned to participants for comment and/or correction? | After the interview, it was mentioned that participants were able to ask for the transcripts. No participants made use of this. |
| **Domain 3: analysis and findings** | | | |
| Data analysis | | | |
| 24. | Number of data coders | How many data coders coded the data? | The first three transcripts were coded by TC, MR and EK. Hereafter transcripts were coded separately by TC and MR. |
| 25. | Description of the coding tree | Did authors provide a description of the coding tree? | The coding tree was deductively and inductively developed. |
| 26. | Derivation of themes | Were themes identified in advance or derived from the data? | Themes were initially derived from the interview protocol and new subthemes were based inductively with the collected data. |
| 27. | Software | What software, if applicable, was used to manage the data? | Nvivo version 12 |
| 28. | Participant checking | Did participants provide feedback on the findings? | No |
| Reporting | | | |
| 29. | Quotations presented | Were participant quotations presented to illustrate the themes / findings? Was each quotation identified? e.g. participant number | Participant quotations were presented to illustrate the findings by using a participant number |
| 30. | Data and findings consistent | Was there consistency between the data presented and the findings? | Yes |
| 31. | Clarity of major themes | Were major themes clearly presented in the findings? | Yes |
| 32. | Clarity of minor themes | Is there a description of diverse cases or discussion of minor themes? | Yes |

# Appendix 3: Coding tree


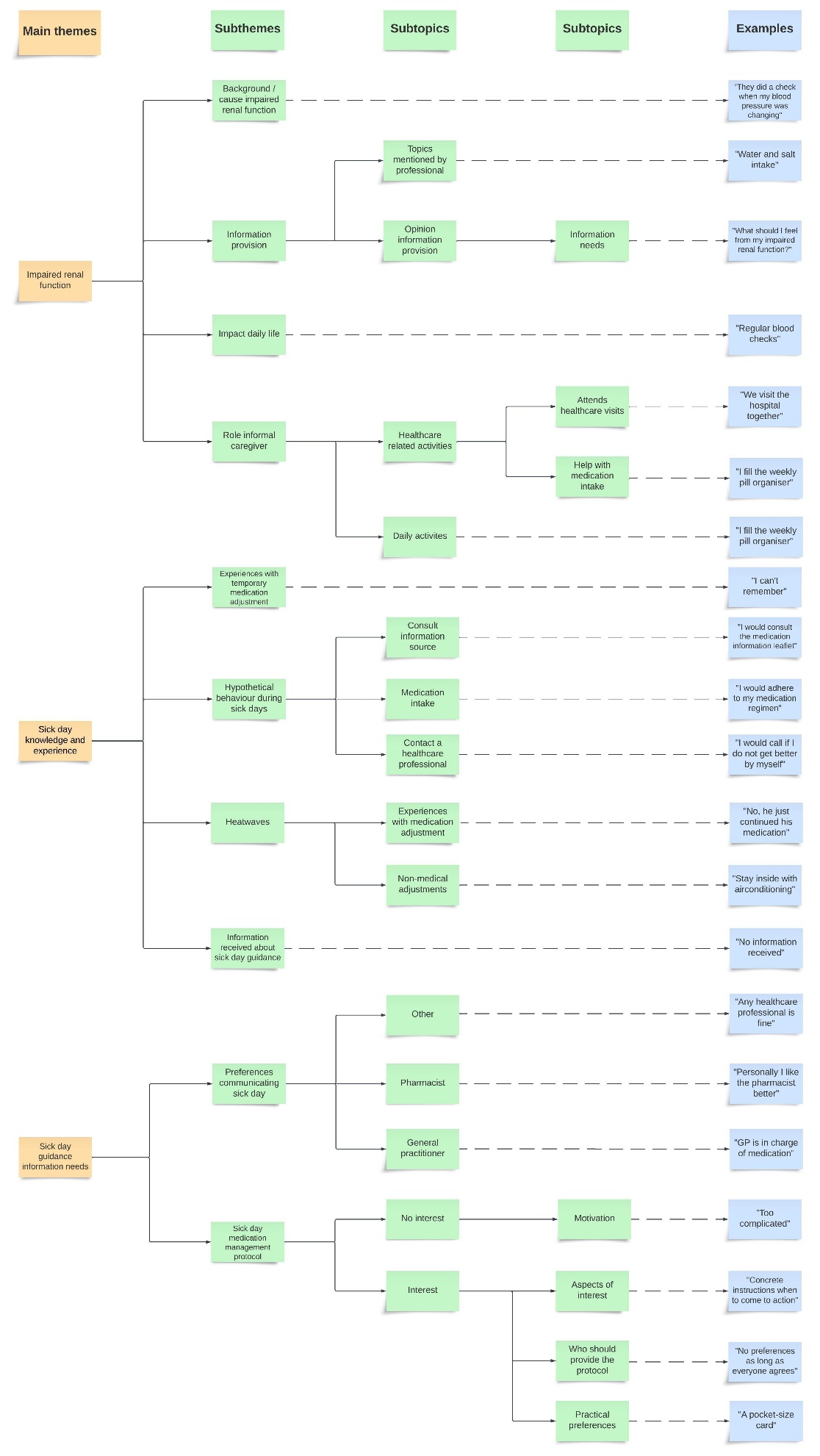

Supplement: Supplemental Material [file IGEN_A_2413097_SM4983.docx]
